# Comprehensive Molecular Diagnostic Tests in Non-Small Cell Lung Cancer: Frequency of *ALK*, *ROS1*, *RET*, and Other Gene Fusions/Rearrangements in a Romanian Cohort

**DOI:** 10.3390/cancers17223673

**Published:** 2025-11-17

**Authors:** Ester-Andreea Cohn (Vizitiu), Ecaterina Tataru, Ortansa Csutak

**Affiliations:** 1 Faculty of Biology, University of Bucharest, 050095 Bucharest, Romania; 2 Department of Molecular Biology and Genetics, GRAL Medical, 031424 Bucharest, Romania; ecaterina.tataru@gralmedical.ro; 3 Department of Genetics, Faculty of Biology, University of Bucharest, 060101 Bucharest, Romania; ortansa.csutak@bio.unibuc.ro

**Keywords:** lung, cancer, *NSCLC*, *NGS*, *ALK*, *ROS1*, *RET*, *NTRK*, fusions

## Abstract

**Background/Objectives**: Lung cancer remains among the most frequently diagnosed malignancies in Romania, with a high mortality rate. Beyond *EGFR* mutations, clinically relevant genetic alterations in non-small cell lung cancer (NSCLC) include fusions involving *ALK*, *ROS1*, *RET*, and *NTRK1/2/3*. This study aimed to determine the prevalence of these mutations in a Romanian cohort and evaluate their associations with clinicopathological features. **Methods**: DNA and RNA were simultaneously extracted from formalin-fixed, paraffin-embedded (FFPE) tissue sections using the Genexus Purification System (ThermoFisher Scientific). Concentrations were quantified fluorometrically, and gene fusions were analyzed with Ion Torrent NGS (Ion GeneStudio S5) with the Oncomine Focus Assay (ThermoFisher Scientific). Library preparation was automated with the Ion Chef System, and data interpretation was conducted using Ion Reporter. **Results**: Among 721 newly diagnosed NSCLC patients, 28 (3.88%) harbored gene fusions. Adenocarcinoma prevailed among fusion-positive cases (85.7%). The subgroup included 15 males and 13 females, with a mean age of 63.25 years (range 43–83). *ALK* fusions were most frequent (1.66% of the cohort; 42.86% of positives), predominantly *EML4::ALK*. *ROS1* fusions were detected in five patients (0.7%), most frequently *CD74::ROS1*. *RET* fusions occurred in 1.11%. Rare fusions included one *ETV6::NTRK3*, one *PTPRZ1::MET*, and one *FGFR3::TACC3* co-occurring with *EGFR L858R*. **Conclusions**: Gene fusions were present in a minority of NSCLC cases, with *ALK*, *ROS1*, and *RET* being the most clinically relevant. These alterations were mutually exclusive with common drivers such as *EGFR* or *KRAS*. Detection of rare fusions highlights the therapeutic potential of comprehensive NGS profiling in Romanian NSCLC patients.

## Simple Summary

Lung cancer remains one of the leading causes of cancer-related death worldwide. Molecular testing plays an essential role in detecting specific genetic alterations that can guide targeted therapy, especially in non-small cell lung cancer (NSCLC). In this study, we analyzed a cohort of newly diagnosed, treatment-naïve, non-squamous and non-smoking squamous NSCLC patients from Romania, particularly to assess the presence of gene fusions involving *ALK*, *ROS1*, *RET*, and *NTRK*, and, additionally, less common genes such as *FGFR3* and *MET*. These gene fusions were present in a relatively small percentage of cases, supporting the need for comprehensive molecular profiling in both non-squamous and squamous NSCLC, particularly among non-smokers. Overall, this study emphasizes the value of broad genetic testing for improving diagnosis and opening new directions for personalized therapy.

## 1. Introduction

Lung cancer remains one of the leading causes of cancer-related mortality worldwide. Histologically, it is classified into two major types: non-small cell lung cancer (NSCLC) and small cell lung cancer (SCLC), which together account for approximately 95% of all diagnosed cases. NSCLC includes several histological subtypes, most commonly adenocarcinoma, squamous cell carcinoma, adeno-squamous carcinoma, and large-cell carcinoma. In Romania, a 2025 study conducted in Timiș County reported that lung cancer cases nationwide increased by 80% over the past 5 years [1].

Historically, systemic chemotherapy was the primary therapeutic option available for lung cancer patients. Advances in molecular oncology have facilitated the understanding of tumorigenesis, leading to the development of targeted therapies. In NSCLC, commonly tested molecular markers with therapeutic relevance include *EGFR*, *ALK*, *KRAS G12C*, *ROS1*, *BRAF V600E*, *NTRK1/2/3*, *MET*, *RET*, and *ERBB2*. Detection of alterations in these genes can be performed using next-generation sequencing (NGS), real-time PCR, fluorescence in situ hybridization (FISH), or immunohistochemistry (IHC), depending on the molecular particularities of each target [2,3].

With the evolution of sequencing technologies, gene fusions—resulting from chromosomal rearrangements—have emerged as clinically relevant oncogenic alterations. Gene fusions can be found in a variety of cancers, including hematologic malignancies such as chronic myeloid leukemia, as well as solid tumors such as those of the lung, breast, prostate, soft tissue, and brain. An analysis of a data set from The Cancer Genome Atlas (TCGA) identified 20,731 gene fusions across 10,000 tumor samples, encompassing 33 cancer types. Another large-scale analysis of 9624 tumor tissue samples reported 25,664 gene fusions, suggesting that gene fusions contribute to tumorigenesis in approximately 16.5% of cases and act as initiating drivers in more than 1% [4,5,6].

These findings underscore the critical role of gene fusions in the pathogenesis and targeted treatment of NSCLC. *ALK* gene fusions are the most frequently identified in NSCLC patients, followed by *ROS1* and *RET* fusions. Although fusions involving *NTRK*, *NRG1*, *FGFR*, *MET*, *EGFR*, and *BRAF* are rare, they may still have important therapeutic implications.

International guidelines recommend extended molecular testing for patients with locally advanced or metastatic NSCLC, including assessments of *ALK*, *ROS1*, *RET*, and *NTRK1/2/3* gene fusions. Targeted therapy with specific tyrosine kinase inhibitors (TKIs) has become a standard first-line therapy for stage IV fusion-positive NSCLC, underscoring the need for timely and accurate molecular diagnosis to ensure optimal therapeutic strategies [7,8].

The present article aims to determine the prevalence of the most clinically relevant gene fusions/rearrangements in NSCLC in the Romanian population and evaluate the feasibility of using this method to identify patients eligible for targeted therapies, emphasizing the need for broader implementation of NGS in routine clinical practice in Romania

## 2. Materials and Methods

### 2.1. Patient Sampling

The current study analyzed 721 formalin-fixed, paraffin-embedded (FFPE) tissue samples obtained from patients newly diagnosed with squamous or non-squamous NSCLC in the metastatic or locally advanced stage, tested at GRAL Medical clinic (Bucharest, Romania) as part of Romania’s National Oncological Patient Testing Program conducted in 2024. The patient cohort comprised exclusively Caucasian non-smoking individuals distributed across the country. Patients were stratified by age and gender.

All samples included in the present study underwent an initial evaluation by a pathologist to establish or confirm the histopathological diagnosis and to determine the tumor cell percentage. According to the pathologist’s recommendations, the tumor cellularity should exceed 20–25%. However, considering that samples from patients with NSCLC predominantly consisted of fine-needle biopsy specimens, samples with lower cellularity were also included in the analysis. Additionally, metastatic tissue samples, including those from bone, liver, and brain, were assessed. The study was conducted in compliance with the Declaration of Helsinki and received approval from the GRAL Medical Clinic’s ethics committee.

### 2.2. DNA and RNA Purification for NGS Testing

FFPE tissue samples underwent deparaffinization and protease digestion for the extraction of DNA and RNA. For nucleic acid purification, the Genexus™ Purification System (ThermoFisher Scientific, Waltham, MA, USA) was employed, using MagMAX™ (ThermoFisher Scientific, Waltham, MA, USA) technology based on magnetic particles with enhanced nucleic acid-binding capacity. This method is suitable for samples of different viscosities and allows elution in a reduced volume. The system incorporates an onboard Qubit™ Fluorometer (ThermoFisher Scientific, Waltham, MA, USA) for automated quantification [9,10]. The platform allows the simultaneous extraction of twelve samples, with separate elution of nucleic acids. The Oncomine Focus Assay kit requires a total input of 10 ng of DNA and RNA, respectively, for library preparation.

### 2.3. NGS Using Ion Torrent Technology

NGS was performed using the Ion Torrent next-generation sequencing (NGS) technology, using the Ion Chef System (ThermoFisher Scientific, Waltham, MA, USA) for automated library preparation and sequencing chip loading (templating process), in conjunction with the Ion GeneStudio S5 System (ThermoFisher Scientific, Waltham, MA, USA). Library preparation was performed using Ion AmpliSeq (ThermoFisher Scientific, Waltham, MA, USA), while the Oncomine™ Focus Assay, Chef-Ready Kit (ThermoFisher Scientific, Waltham, MA, USA) provided primer sets for DNA and RNA libraries, respectively. The DNA primer pools require 10 ng of total genomic DNA (gDNA), at a minimum concentration of 0.67 ng/µL for DNA library preparation.

In the case of RNA samples, complementary DNA (cDNA) synthesis was performed prior to library preparation using the Ion Torrent™ NGS Reverse Transcription Kit (ThermoFisher Scientific, Waltham, MA, USA). The RNA primer pool requires 10 ng of total RNA, at a minimum concentration of 0.8 ng/µL, for RNA library preparation. Prepared libraries were quantified using the Qubit™ Fluorometer to verify the concentration.

RNA and DNA libraries were equalized to 100 pM using the Ion Chef Instrument (ThermoFisher Scientific, Waltham, MA, USA). A combined library was prepared at a 4:1 DNA:RNA ratio, with a final concentration of 33 pM. Eight DNA samples and eight RNA samples were loaded onto an Ion 520 Chip, which has a throughput of 4–6 million reads (Ion 520 Chip Kit, ThermoFisher Scientific, Waltham, MA, USA). Sequencing was performed on an Ion S5 Plus instrument (ThermoFisher Scientific, Waltham, MA, USA) [11].

#### 2.3.1. QC Metrics

All NGS analyses were performed in a clinical laboratory that participated in regular external quality assessment (EQA) schemes for lung cancer, verifying the accuracy, reproducibility, and reliability of results. Following the initial method validation, several sequencing runs were performed, including samples with previously known positive results, to ensure the accuracy and reliability of the assay. Also, NTC (no template control) was tested in each sequencing run to exclude the possibility of contamination.

**Figure 1:**
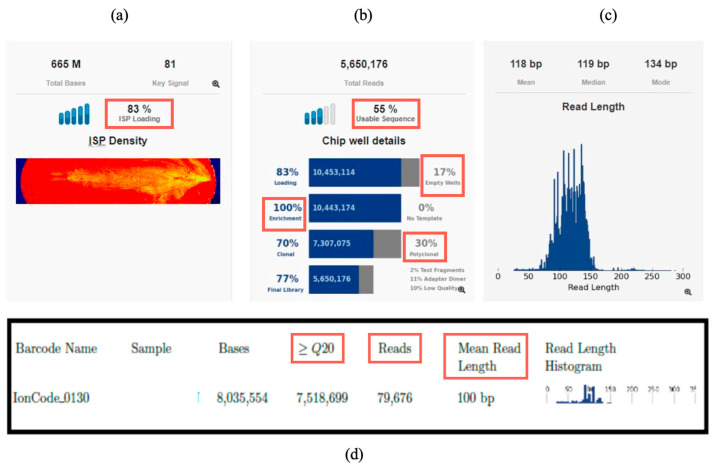
Run parameters overview in Torrent Suite Software Version 5.20: (**a**) Chip loading (ISP loading); (**b**) usable sequence percent, empty wells percent, enrichment percent, and polyclonal and low-quality sequences percent; (**c**) read length histogram; (**d**) example of metrics reported for one RNA sample: Reads, mean read length, Q20.

NGS runs were processed with Torrent Suite Software Version 5.20 (Thermo Fisher Scientific, Waltham, MA, USA), and quality metrics were evaluated according to Oncomine Focus Assay specifications. Acceptable parameter values included ISP loading of 80–90%, clonal ISP rates > 45%, enrichment near 100%, polyclonal ISPs < 45%, low-quality reads < 20%, and usable reads of 50–60% (Figure 1a,b). High sequencing accuracy was confirmed by the Q20 metric (>80% of bases with ≤1% error probability), ensuring reliability for fusion and variant analyses (Figure 1d).

The QC metrics were assessed to confirm gene fusions, including fusion counts, number of reads, read counts per million, imbalance score, validity of expression control, and mean read length. For a positive result of a gene fusion, the count of reads must exceed 20, but, simultaneously, all the QC metrics must also be met.

In the Torrent Suite Run Summary report, we assessed that each sample generated sufficient sequencing reads for each individual sample, both at the DNA and RNA level (20,000–150,000 reads for RNA). In addition, for RNA, the mean read length parameter was assessed, and it consistently exceeded 60 bp (Figure 1c,d). These quality parameters ensured robust input data, allowing gene fusion detection and annotation in Ion Reporter with high confidence.

#### 2.3.2. Data Analysis

**Figure 2:**
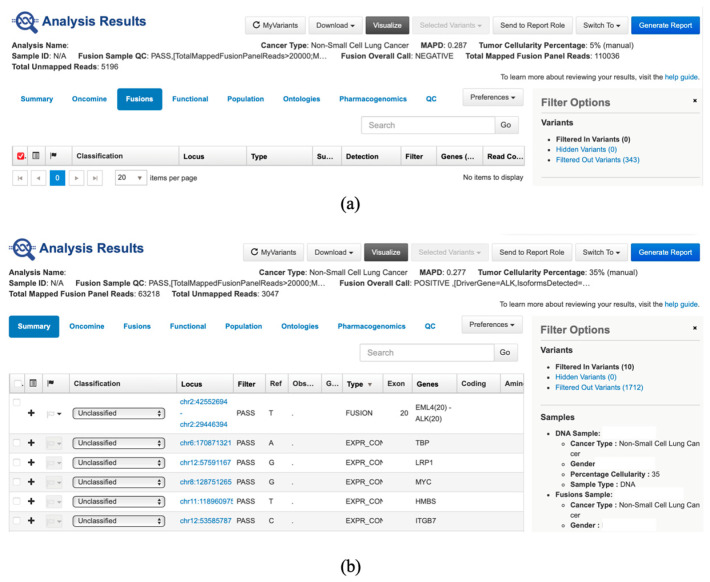
(**a**) Presentation of fusion negative result in Ion Reporter Software (can be observed that the fusion sample QC is passed, fusion overall call is negative and total mapped fusion panel reads is above 20,000); (**b**) Presentation of fusion-positive result in Ion Reporter Software (can be observed that the fusion sample QC is passed, fusion overall call is positive (fusion *EML4(20)::ALK(20)* was identified with a read count of 6152 (>20), and total mapped fusion panel reads is above 20,000).

Sequencing data from the Ion Torrent platform were processed in Torrent Suite Software Version 5.20 (Thermo Fisher Scientific, Waltham, MA, USA) and analyzed in Ion Reporter Software Version 5.20 using the Fusion workflow. All samples passed quality control for fusion detection. Fusions were classified as negative or positive (Figure 2a,b). Only runs with more than 20,000 total mapped reads, and known fusions with more than 20 supporting reads, were considered reportable. Our results were evaluated by ThermoFisher experts and through external quality control assessments, confirming that the data are valid and of high quality. We are currently taking steps to integrate RNA integrity number (RIN) assessment into the workflow.

#### 2.3.3. Variant Annotation and Results Reporting

The *Oncomine Focus Assay* detects hotspot mutations in 35 genes, copy number variations (CNVs) in 19 genes, and gene fusions in 23 target genes (Table 1). Regarding gene fusions, both intergenic and intragenic fusions can be detected, including known fusions, novel fusions, and gene imbalances [12].

**Figure 3:**
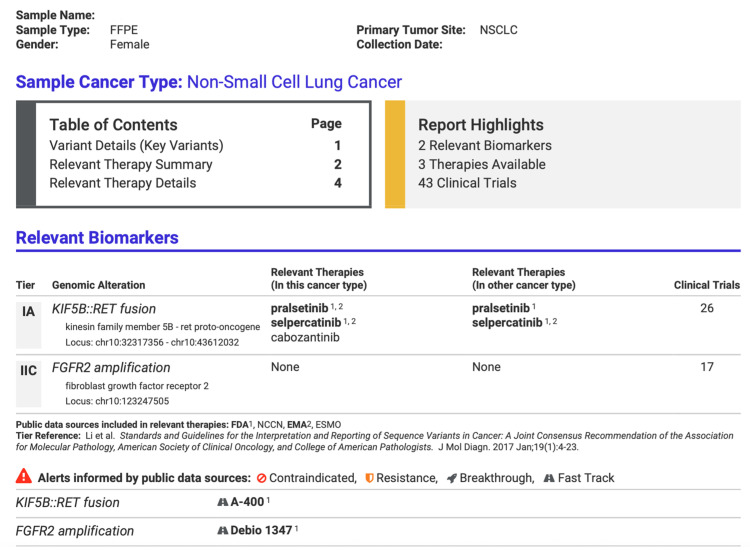
Part of a customized report from Oncomine Reporter Software (ThermoFisher Scientific, Waltham, MA, USA) from a positive patient identified with gene fusion *KIF5B::RET*, summarizing potential targeted therapies.

Variant analysis was performed using Ion Reporter Software Version 5.20 (ThermoFisher Scientific, Waltham, MA, USA) in conjunction with Oncomine Reporter Software Version 6.1 (ThermoFisher Scientific, Waltham, MA, USA), linking each variant to relevant published evidence. Customized PDF reports summarized identified gene fusions and their potential clinical significance (Figure 3). This report was used only as an additional tool. It should be noted that the clinical decisions are the responsibility of the treating oncologist.

## 3. Results

**Figure 4:**
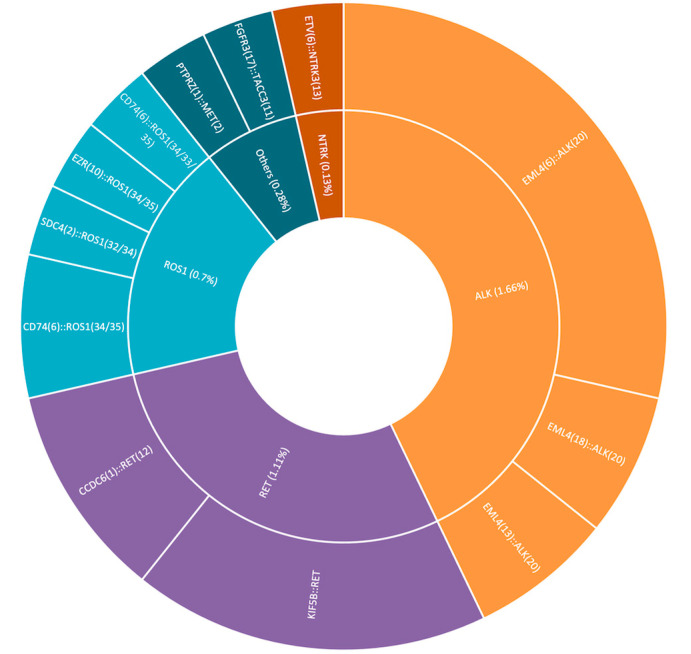
Fusion-positive case distribution.

A cohort of 721 tested patients was analyzed as part of Romania’s National Oncological Patient Testing Program at the GRAL Medical Molecular Biology Laboratory during 2024. All patients included in the study were non-smokers who were newly diagnosed with either squamous or non-squamous non-small cell lung cancer (NSCLC) in metastatic or locally advanced stages (clinical stage III/IV). The primary molecular targets were clinically relevant gene fusions in NSCLC, including *ALK*, *ROS1*, *RET*, and *NTRK1/2/3*, as well as MET and FGFR3, which are not currently part of the recommended national testing panel, as studies in the scientific literature have reported their potential association with NSCLC and therapeutic relevance [4,13,14]. Within the tested cohort, 28 patients (3.88% of the cohort) were identified as positive for gene fusions involving *ALK*, *ROS1*, *RET*, *NTRK*, *MET*, or *FGFR3*. The distribution of fusion-positive cases is presented in Table 2, Figure 4. Among the clinicopathological characteristics, key demographic parameters such as age and gender were assessed (Table 3). Additional variables, including detailed tumor characteristics and molecular correlations, will be further investigated in future studies.

Adenocarcinoma was identified as the prevailing histopathological diagnosis in 24 of the 28 fusion-positive confirmed patients (85.7%), followed by squamous cell carcinoma, observed in 3 patients (10.7%). Additionally, one patient with pleomorphic lung carcinoma was identified.

## 4. Discussion

The study cohort was obtained from January to December 2024, concurrent with the implementation of Romania’s National Oncological Patient Testing Program. This program includes molecular testing panels targeting different cancer types, including non-small cell lung cancer. For NSCLC patients, molecular testing panels are offered based on clinical context and may include *EGFR* mutation analysis by real-time PCR, *ALK* and *PD-L1* testing by immunohistochemistry, or testing for EGFR (exons 18–21) and ALK, ROS1, RET, NTRK1/2/3 fusions by next-generation sequencing. For the NSCLC gene fusion testing, patient inclusion criteria were based on current evidence, selecting non-squamous and non-smoking squamous, newly diagnosed, locally advanced, or metastatic (clinical stage III/IV) stages [15].

The mean age of the fusion-positive subgroup was 63.25 years, with a range from 43 to 83 years and a standard deviation of ±11.2 years. Regarding smoking status, all patients were non-smokers.

Among the 28 fusion-positive patients included in the study, 12 presented *ALK* gene fusions, representing approximately 1.66% of the total cohort of 721 tested patients. The most frequently identified fusion was *EML4::ALK*, observed in 12/12 cases (1.66% of the total tested cohort; 42.86% of the fusion-positive subgroup), which was consistent with data reported in the scientific literature, although the patient cohort in this study was smaller [16,17]. Only one patient harboring this fusion was diagnosed with non-keratinizing squamous cell carcinoma, while the remaining patients had adenocarcinoma. A gender-based comparison of *EML4::ALK* fusion prevalence revealed four female and eight male patients, suggesting a higher prevalence among males (66.67%). The scientific literature indicates a higher prevalence of the *EML4::ALK* fusion among female patients, particularly in Asian populations, where larger cohorts have been tested and analyzed [17]. The mean age of patients harboring the *EML4::ALK* fusion was 62 years. Other existing studies, particularly those involving Asian female populations, show a tendency for a younger mean age among *EML4::ALK* patients (50–60 years) [3]. The observed mean age in this study is situated at the upper end of this range, which may suggest slightly older mean ages in Western or mixed-population cohorts [18].

As the national testing program requires fusion testing to be performed exclusively in newly diagnosed patients, the occurrence of these mutations as mechanisms of acquired resistance to TKI could not be evaluated in this cohort. However, it is notable that no co-occurrence of the *EML4::ALK* fusion with *EGFR* mutations was observed, confirming the mutual exclusion of the two key driver alterations in NSCLC [3,19].

Five of the 28 fusion-positive patients exhibited *ROS1* gene fusions (0.7% of the total cohort of 721 patients). The most frequently identified fusion was *CD74::ROS1*, detected in three out of the five cases (0.4% of the total cohort; 10.7% of the fusion-positive subgroup), in line with existing data in the scientific literature [20]. Among the patients with this fusion, histopathological diagnoses included one case of non-keratinizing squamous cell carcinoma, one pleomorphic lung carcinoma, and one adenocarcinoma. The mean age of *ROS1* fusion-positive patients was 62.6 ± 13.6 years, and three of the five patients were male. Due to the small sample size, these findings should be interpreted with caution and cannot be generalized. 

*RET* gene fusions were identified in eight patients, accounting for 1.11% of the total cohort. The most frequent being the *KIF5B::RET* fusion, observed in five patients (approximately 0.7% of the total tested cohort; 17.86% of the fusion-positive subgroup), which is consistent with previously published data [21,22,23]. The remaining three cases carried the *CCDC6::RET* fusion (0.41% of the total cohort of 721 patients). Adenocarcinoma was again the predominant histopathological subtype, observed in all eight cases. The *RET* fusion-positive cohort consisted of eight patients, evenly distributed by sex (four males and four females), with a mean age of 70.9 ± 11.8 years. Due to the small sample size, these findings should be interpreted with caution and cannot be generalized.

Among the 721 patients included in the study, a single case of *NTRK3* gene fusion (*ETV6::NTRK3*) was identified (0.13% of the total cohort) [24,25]. The patient was a 65-year-old female diagnosed with pulmonary adenocarcinoma, with no prior treatment using tyrosine kinase inhibitors (TKIs) and no co-occurring mutations associated with sensitivity to such therapies. The *ETV6::NTRK* fusion is extremely rare in NSCLC patients, and its detection by immunohistochemistry (IHC) may yield highly heterogeneous or even false-negative results [26].

One case involving a MET gene fusion, specifically *PTPRZ(1)::MET(2),* was identified (female patient, age 74), as well as one case of *FGFR3:TACC3* fusion (the most common *FGFR* fusion reported in NSCLC) co-occurring with the *EGFR L858R* mutation (female patient, age 72) [27].

*FGFR* gene fusions have been reported in the literature in approximately 8–10% of solid tumors and around 0.2% of NSCLC cases, with *FGFR3::TACC3* being the most frequently identified variant. In a large German study analyzing RNA-based NGS data from 3309 NSCLC patients, 21 *FGFR3::TACC3*-positive cases (0.63%) were identified, predominantly among male patients with a median age of 69 years, a higher prevalence of squamous cell carcinoma, and favorable responses to immunotherapy. In comparison, our cohort showed a lower prevalence (0.14%), likely due to differences in sample size, smoking status, and tumor subtype composition. The co-occurrence of *FGFR3::TACC3* and *EGFR L858R* mutation is rare and has been only sporadically reported in the literature. Most reports describe *FGFR3::TACC3* as an acquired event after *EGFR*-TKI therapy, and its presence in a TKI-naïve tumor suggests a potential de novo co-activation of independent oncogenic pathways. Although the biological implications remain unclear, simultaneous activation of the *EGFR* and *FGFR* signaling axes could contribute to tumor heterogeneity and influence therapeutic response. This finding underscores the importance of comprehensive NGS profiling at initial diagnosis to identify uncommon but clinically relevant co-alterations and highlights the need for future large-scale studies and the inclusion of gene fusion testing for smokers and patients treated with *EGFR* TKIs within Romania’s national molecular testing program [13,27,28,29].

The *PTPRZ1::MET* fusion has been reported in the literature in only a few isolated cases, is most often identified in brain metastases from NSCLC rather than in primary tumors, and is predominant in patients previously treated with TKIs. The fusion leads to increased *MET* expression, constitutive activation of the *MET* kinase (high levels of phosphorylation), and an aggressive oncogenic phenotype. In the present study, the patient harboring this alteration was tested from a treatment-naïve primary tumor, highlighting the novelty and potential clinical significance of detecting this rare fusion at initial diagnosis [30,31].

Both genes also exhibit alterations with implications in NSCLC, represent therapeutic targets, and have been associated with the development of resistance to tyrosine kinase inhibitors. Previous studies have reported the co-occurrence of *FGFR* gene fusions and *EGFR* mutations, particularly in patients previously treated with tyrosine kinase inhibitors, suggesting a potential role of *FGFR* fusions as a resistance mechanism. In the present study, the overlap of these alterations was confirmed; however, given that all patients were newly diagnosed and treatment-naive, the finding cannot be attributed to acquired resistance [4,32,33,34].

Adenocarcinoma is established as the most prevalent histological subtype of NSCLC in individuals who have never smoked. In this study, the predominance of this histopathological subtype likely reflects that the cohort consisted exclusively of non-smokers. This finding confirms the existing data in the literature, which reports a significantly higher incidence of adenocarcinoma in non-smoking populations compared to other NSCLC subtypes [35,36]. It is important to mention that the current study has some limitations. Although the patient cohort included individuals referred from multiple regions across the country, the study is a single-center analysis, which may limit the applicability of the findings. Also, the restriction of the inclusion criteria to newly diagnosed, non-smoking NSCLC patients may lead to an underestimation of the gene fusions in the broader population (TKI-treated or smokers). Several studies have demonstrated that gene rearrangements such as ALK, ROS1, RET, and NTRK can also occur, although at lower frequencies, among smokers and in patients with previously treated or recurrent disease [4,36,37]. Clinical outcomes (treatment choice and response, progression-free survival, overall survival, etc.) were not assessed, limiting conclusions on the therapeutic impact of the identified alterations.

In Romania, large-scale implementation of NGS remains limited by unequal access to molecular testing, variable reimbursement policies, and the high upfront costs of equipment, reagents, and bioinformatics support. Most analyses are still performed in tertiary centers, and the number of laboratories performing such tests nationwide remains low, even after the launch of the national molecular testing program. This emphasizes the need to increase awareness of the clinical value of NGS and to promote its broader adoption through coordinated investment in infrastructure, personnel training, and standardized data pipelines. Comprehensive genomic profiling can be cost-effective in the long term by reducing sequential single-gene testing and guiding targeted therapies that improve patient outcomes. In lung cancer, where actionable alterations such as *EGFR*, *ALK*, *ROS1*, and *MET* have direct therapeutic implications, optimizing patient selection for NGS testing could further enhance cost-efficiency. Broader characterization of Romanian NSCLC patients may thus refine testing criteria and stimulate both public and private initiatives toward nationwide implementation of precision oncology.

## 5. Conclusions

Clinically relevant gene fusions, including *ALK*, *ROS1*, *RET*, *NTRK*, *FGFR3*, and *MET*, occur in a small yet clinically meaningful subset of non-smoking NSCLC patients, underscoring the value of broad NGS-based molecular profiling. The mutual exclusivity of *ALK* rearrangements and EGFR mutations supports current targeted therapy strategies. Implementation of Romania’s National Oncological Molecular Testing Program has enabled early detection of actionable alterations, highlighting the potential benefit of expanding testing to a broader patient population. Future studies with larger, more diverse cohorts that integrate clinical outcomes are warranted. These studies could better define the prevalence, clinical impact, and cost-effectiveness of comprehensive fusion testing in NSCLC.

## Figures and Tables

**Table 1:** Oncomine Focus Assay target genes.

Hotspot Genes	Copy Number Variation (CNV) Genes	Fusion Genes
*AKT1*, *FGFR2/3*, *MET*, *ALK*, *GNA11*, *MTOR*, *AR*, *GNAQ*, *NRAS*, *BRAF*, *HRAS*, *PDGFRA*, *CDK4*, *IDH1/2*, *PIK3CA*, *CTNNB1, RAF1*, *DDR2*, *JAK1/2/3*, *RET*, *EGFR*, *ROS1*, *ERBB2/3/4*, *SMO*, *KIT*, *KRAS*, *ESR1*, *MAP2K1/K2*	*ALK*, *KRAS*, *AR*, *MET*, *BRAF*, *MYC*, *CCND1*, *MYCN*, *CDK4/6*, *PDGFRA*, *PIK3CA*, *EGFR*, *ERBB2*, *FGFR1/2/3/4*, *KIT*	*ABL1*, *FGFR1/2/3*, *ALK*, *AKT3*, *MET*, *AXL*, *NTRK1/2/3*, *BRAF*, *EGFR*, *ERBB2*, *PDGFRA*, *ERG*, *PPARG*, *ETV1/4/5*, *RAF1*, *RET*, *ROS1*

**Table 2:** Fusion-Positive Case Counts.

*EML4-ALK*		*ROS1*	
***EML4(6)::ALK(20)***	8	*CD74(6)::ROS1(34) + CD74(6)::ROS1(35)*	2
***EML4(13)::ALK(20)***	2	*CD74(6)::ROS1(34) + CD74(6)::ROS1(33) + CD74(6):ROS1(35)*	1
***EML4(18)::ALK(20)***	2	*CD74(6)::ROS1(34) + CD74(6)::ROS1(33) + CD74(6):ROS1(35)*	1
		*SDC4(2)::ROS1(32) + SDC4(2)::ROS1(34)*	1
***RET***		*Others*	
***KIF5B::RET***	5	*FGFR3(17)::TACC3(11)*	1
***CCDC6(1)::RET(12)***	3	*PTPRZ(1)::MET(2)*	1
		*ETV(6)::NTRK3(11)*	1

**Table 3:** Attributes of the fusion-positive patients.

Parameters	Number of Patients (Total = 28)
Sex—*n* (%)	
Male	15 (53.57%)
Female	13 (46.42%)
Age-mean (±SD)—*n* = 28	
30–45	3
46–60	5
61–75	14
>75	6
Smoking status—*n* (%)	
Never smoker	28 (100%)
EGFR status—*n* (%)	
Negative (Wild-Type)	27 (99%)
Positive	1 (1%)—*L858R* (co-occurring with *FGFR3::TACC3* fusio)

## Data Availability

The datasets used and/or analyzed during the current study are available from the corresponding author on reasonable request.
